# Comprehensive methodology for standardized ecotoxicological assessment of TiO_2_-based sunscreen leachates in aquatic environment

**DOI:** 10.3389/ftox.2025.1686954

**Published:** 2025-11-03

**Authors:** Roberta Nugnes, Giulia De Negri Atanasio, Elisabetta Perata, Erica Lertora, Lorenzo Dondero, Federica Robino, Francesca Tardanico, Cristina Capelli, Fabio Ghioni, Tania Cai, Dalia Gobbato, Norina Marciani, Roberta Miroglio, Matteo Zanotti Russo, Veronica Piazza, Marco Faimali, Chiara Gambardella, Francesca Garaventa, Elena Grasselli

**Affiliations:** ^1^ Institute for the Study of Anthropic Impact and Sustainability in the Marine Environment, National Research Council, Genova, Italy; ^2^ Department of Earth, Environment and Life Science, University of Genoa, Genova, Italy; ^3^ Interuniversity Center for the Promotion of 3R Principles in Teaching and Research (Centro 3R), Pisa, Italy; ^4^ Angel Consulting S.a.s, Milano, Italy; ^5^ Regional Environmental Protection Agency, Liguria, Italy; ^6^ National Center for the Development of New Technologies in Agriculture (Agritech), Napoli, Italy; ^7^ Biostructures and Biosystems National Institute, Roma, Italy

**Keywords:** algae, crustacean, ecotoxicity, marine pollution, freshwater pollution, sunscreen, titanium dioxide

## Abstract

**Introduction:**

This study evaluates the ecotoxicity of micro- and nano-sized titanium dioxide (TiO_2_), either as active ingredients or incorporated into sunscreen formulations in the aquatic environment, by proposing a leaching protocol simulating a realistic scenario of human immersion in freshwater and seawater.

**Methods:**

To this aim, an ecotoxicological screening of micro- and nano-TiO_2_ active ingredients and incorporated into sunscreens was applied, by evaluating acute and sub-acute responses (bioluminescence and growth inhibition, immobilization, behaviour) in freshwater and marine bacteria, microalgae and crustaceans. Then, Ti concentration was measured in the leachates of sunscreens through Inductively Coupled Plasma mass spectrometry (ICP-MS).

**Results and discussion:**

Toxic effects (EC_50_s) were only found in microalgae and crustaceans exposed to TiO_2_ active ingredients. No toxicity occurred with sunscreens formulations, although significant algal growth inhibition was determined, likely due to TiO_2_ size rather than Ti concentration. By integrating a sunscreen leachate based methodology with a multi-species and multi-endpoint approach, this study introduces a novel ecosafety-oriented assessment of TiO_2_ providing realistic ecotoxicological evidence relevant to freshwater and marine environments.

## 1 Introduction

In 2015, the United Nations Member States adopted the 2030 Agenda for Sustainable Development, designed to serve as both a roadmap and a plan of action for achieving peace and prosperity through the fulfilment of 17 Sustainable Development Goals (SDGs). Specifically, SDG six focuses on ensuring the availability and sustainable management of water and sanitation (UN General Assembly, 2015). Moreover, the UN Water report (2018) highlights that the quality and availability of water are necessary for the improvement of society, environmental health, and economic wealth. However, it is widely recognized that the aquatic ecosystem is profoundly affected by the presence of emerging contaminants (Rathi et al., 2021; Prajapati et al., 2023; Sultan et al., 2024; Ghosh et al., 2024). Pharmaceutical compounds, micro- and nanoplastics, pesticides, nanomaterials and personal care products included cosmetics, skin and hair care products, household cleaners, and sunscreen are considered some of the most concerning pollutants (Barreto et al., 2023; Sudarsan et al., 2024).

In this scenario, the increasing awareness of the negative effects of solar radiation on human skin such as reactive oxygen species production, photoaging, DNA damage and skin cancer (Tang et al., 2024) has led to a heightened use of sunscreens (Heerfordt et al., 2017; Zou et al., 2020). Ultraviolet (UV) filters, contained in sunscreens and responsible for protecting the skin (Sabzevari et al., 2021), are divided into organic and inorganic compounds. The first ones (i.e., octocrylene and benzophenone-3) absorb UV light, while the second ones (i.e., TiO_2_ or ZnO) reflect and scatter UV light (Ramos et al., 2016). Nowadays, the number of UV filters approved for use in sunscreens depends on the geographical area: 29 in Europe, 16 in the United States, 22 in Canada, 27 in China and 31 in Australia. Nevertheless, their concentration in sunscreens always varies from 0.1% to 10% (Lavorgna et al., 2024).

The European Cosmetic Product Regulation 1223/2009 stands as a comprehensive framework governing the production and market placement of cosmetic products within the European Union. Its core premise is grounded in the belief that all cosmetics must be inherently safe for their normal and reasonably foreseeable use. This regulation, while not prescriptive in terms of permissible ingredients, relies on a structured system consisting of five annexes to enforce its provisions. These annexes comprise a list of forbidden ingredients and another set outlining restrictions on certain substances. Among these annexes, Annex V plays a critical role in regulating the use of UV-filters within cosmetic products, a vital aspect of skin protection.

The integration of ingredients into these annexes is contingent upon the issuance of an opinion by the Scientific Committee on Consumer Safety (SCCS), underscoring the importance of rigorous scientific assessment in cosmetic product safety. The EU Regulation specifically defines “UV-filters” as substances predominantly intended for shielding the skin against specific UV radiation, either through absorption, reflection, or scattering of UV rays.

The annual production of UV filters for the global market is estimated at around 10,000 tons (Couselo-Rodríguez et al., 2022), resulting in potential environmental and health risks. Sunscreens and UV filters can enter the environment directly or indirectly. Some of the compounds could leach from the skin directly into the aquatic ecosystem during recreational activities (surfing, snorkeling, diving, swimming). Conversely, the remaining part could release indirectly due to the inefficiency of wastewater treatment plants in removing them (Miller et al., 2021; Duis et al., 2022). According to recent findings, their presence has been detected in both fresh and sea water (lakes, rivers, coastal waters, groundwater, seas, oceans) (Jyoti and Sinha, 2023) at concentrations in the range ng-µg/L up to mg/L in coastal areas (Cadena-Aizaga et al., 2020; Wong et al., 2020; Beiras et al., 2021; Shetty et al., 2023). Several findings describe molecular, biochemical and cellular changes with consequences in physiological function (e.g., growth, swimming, development, reproduction) in freshwater and seawater organisms (Chatzigianni et al., 2022; Keller, 2023). Regarding phytoplankton, UV filters could cause growth inhibition and oxidative stress in marine diatom *Phaeodactylum tricornutum* (Peng et al., 2011; Wang et al., 2016) and freshwater algae *Raphidocelis subcapitata* and *Chlorella vulgaris* (Ozkaleli and Erdem, 2018; Samei et al., 2019; Feizi et al., 2022). About zooplankton, the exposure to UV filters impaired survival, fertility and swimming of the crustaceans (*Daphnia magna, Artemia franciscana*), echinoderm (*Paracentrotus lividus*) developmental anomalies, and swimming speed of rotifer *Brachionus calyciflorus* (Dong et al., 2020; Boyd et al., 2024; Marcellini et al., 2024; Németh et al., 2024; Ortiz-Román et al., 2024).

Among UV filters, titanium dioxide (TiO_2_) is one of the most used (Labille et al., 2020). The global nanomaterials market is valued at approximately $3 trillion, and TiO_2_ ranks among the top-selling materials (Soler de la Vega et al., 2020), with an estimated annual production of 1300 MT (Abdel-Latif et al., 2020). It is also employed in various cosmetic products, such as lip balms, foundations, and day creams, as well as in orthodontic compounds and as food additives. Since 2000, TiO_2_ has been approved by the Scientific Committee on Consumer Safety (SCCS) as a substance authorized for use in sunscreen formulations at a maximum concentration of 25% (SCCS, 2014), except in spray products (Commission Regulation, 2016).

The detection of inorganic compounds, such as TiO_2_ (both nano- and microsized forms), represents a significant analytical challenge due to their natural occurrence in the environment. Generally, current analytical techniques lack the sensitivity to differentiate between naturally occurring and synthetically produced variants (Labille et al., 2020). However, in seawater, UV filters are primarily detected in the surface layer, thereby distinguishing them from naturally occurring substances predominantly found in the water column or sediments. This phenomenon may be attributed to the high salinity of seawater, which reduces the solubility of sunscreen components. Such behaviour has not been observed in the freshwater ecosystem (Tovar-Sánchez et al., 2013).

Generally, limited data on TiO_2_ concentration report values of µg/L in European and American aquatic environments (Menard et al., 2011). Neal et al. (2011) observed a maximum concentration of 6.48 μg/L in English surface waters, while Gondikas et al. (2014) reported a concentration of 4 μg/L in the Old Danube Lake in Vienna. Conversely, higher concentrations ranging from 52 to 86 μg/L were reported in Chinese river surface waters (Shi et al., 2016). About wastewater treatment plants, concentrations varied from 181 to 1233 μg/L in American tributaries (Westerhoff et al., 2011) and from 26.9 to 43.1 μg/L in Chinese tributaries (Shi et al., 2016). Limited data are available regarding the marine environment. According to Boxall et al. (2007), who estimated that the concentration of TiO_2_ released from cosmetics ranged from 24 to 245 μg/L, a concentration of approximately 37.6 μg/L was reported in the coastal waters of Mallorca (Tovar-Sánchez et al., 2013). In the Mediterranean waters near Marseille, TiO_2_ was found at about 20 μg/L, reaching concentrations of up to 900 μg/L (Labille et al., 2020).

Given these environmental concentrations, concerns have arisen regarding the potential ecotoxicological effects of TiO_2_, particularly in the context of cosmetic formulations (Dreno et al., 2019). While organic UV filters such as oxybenzone and octinoxate have already been identified as hazardous to marine ecosystems - leading to their ban in Key West and Hawaii (Suh et al., 2020) - there is a growing need to assess the impact of inorganic UV filters like TiO_2_. In this scenario, to provide experimental data that may enhance the understanding of the cosmetic production field and support future regulations, this study assesses the safety of nano- and micro-sized TiO_2_ as active ingredient alone and incorporated into sunscreens. With this aim, we examined for the first time the impact of TiO_2_-based sunscreen formulations by applying an International Organization for Standardization (ISO) protocol simulating human immersion in the aquatic environment.

Given the growing need to assess the impact of inorganic UV filters like TiO_2_ and to provide data supporting in cosmetic production and future regulations in line with Safe and Sustainable by Design (SSbD) principles, this study applied for the first time an *ad hoc* protocol simulating realistic human immersion and standardized ecotoxicological procedures (i.e., ISO, UNICHIM). The latter represent a novel ecosafety-oriented multi-species and multi-endpoint approach applied to marine and freshwater bacteria, algae and crustaceans.

## 2 Materials and methods

### 2.1 Chemicals

Active principles Parsol TX (CAS: 13,463-67-7; 82%–87%; rutile-TiO_2_ titanium dioxide, and 10.5%–14.5% silicon dioxide) powder (<1000 μm) and Aerodisp W740X (CAS: 13,463-67-7; anatase-TiO_2_) milky-white liquid solution (40 wt%, 1.41 g/cm^3^ density, mean aggregate size ≤100 nm) were purchased from DSM Nutritional Products and Evonik Industries, respectively. Specifically, Parsol TX stock solution was prepared by dissolving powder in ethanol. The latter did not exceed the 0.02% at the highest concentration tested. All test solutions were obtained by diluting Parsol TX and Aerodisp W740X stock solutions with filtered natural sea water (FNSW, salinity 37‰) and artificial freshwater.

### 2.2 Cream leachate preparation

The cream formulations were kindly provided by AHAVA Dead Sea Laboratories and produced in accordance with Good Manufacturing Practices (GMP). Each formulation contained either Parsol TX (micro-sized titanium dioxide) or Aerodisp W740X (nano-sized titanium dioxide) at a final concentration of 5% w/w. In addition, a control formulation without titanium dioxide (referred to as ‘blank’) was included and subjected to the same leaching and testing procedures. Approximately 72 mg of each cream was uniformly applied to a synthetic skin substrate (6 × 6 cm). The resulting dispersions were then tested either undiluted or diluted at a 1:6 ratio, corresponding to leachate cream concentrations of 72 mg/L (3.6 mg/L of nominal TiO_2_; referred to as 100%) and 12 mg/L (0.6 mg/L of nominal TiO_2_; referred to as 16.6%), assuming complete release of the cream into the medium which represents a worst-case scenario for estimating the highest potential toxicity.

### 2.3 Titanium quantification

Titanium was analytically quantified according to the UNI EN ISO 17294-2 (2023) method. Samples were diluted 1:10 with Milli-Q grade water, acidified to 1% with HNO_3_, and analyzed using Inductively Coupled Plasma Mass Spectrometry (ICP-MS; NexION 350D, PerkinElmer), equipped with an ESI PrepFast 2DX autosampler for automated sample and standard preparation and dilution. The ICP-MS system is a triple quadrupole instrument with a collision/reaction cell (collision gas: helium) for interference suppression. Sample introduction was performed via a pneumatic nebulizer with a cyclonic spray chamber. External calibration was carried out using the internal standard method.

### 2.4 Ecotoxicological tests

Testing of nano- and micro-sized TiO_2_, both as an active ingredient alone and as leachates derived from sunscreens, was carried out in accordance with national (UNICHIM) and international (ISO) standardized procedures to ensure consistency and reliability of the results (Bisinicu et al., 2024). Although *A. franciscana* and *D. magna* are among the most tolerant aquatic invertebrates, these standard test organisms are listed in regulations such as the Italian Legislative Decree No. 152/2006. Particularly, *D. magna* is used as the standard bioassay organism by different international scientific bodies (e.g., American Public Health Association) and governmental agencies (e.g., USA Environmental Protection Agency) as reported by Sarma and Nandini (2006). Furthermore, due to their a short life cycle, smaller size, wide distribution, high population density, the lack of need for feeding, anhydrobiotic storage and ready availability, they are widely used in ecotoxicological assessments (Koivisto, 1995; Neumeyer et al., 2015; Cavion et al., 2020; Ahmed, 2023).

#### 2.4.1 Bacteria

The bioluminescence inhibition of the photobacterium *A. fischeri* was determined according to the UNI EN ISO 11348-3 (EN ISO 11348‐3, 2018) test protocol. The lyophilized bacteria were kept at −20 °C prior testing and activated by hydration. Bioassays were carried out in triplicates by exposing *A. fischeri* to Aerodisp W740X (0.1, 1, 14.1, 141, 1410 mg/L) and Parsol TX (0.1, 1, 12.5, 50 mg/L) solutions, and to blank cream, Aerodisp W740X cream and, Parsol TX cream leachates (100% - undiluted leachates, 16.6% - diluted leachates). All the samples were kept on a thermostatic plate at 15 °C throughout the entire test. Negative and solvent control were performed by NaCl solution and ethanol, respectively. Bioluminescence was measured after 30 min of exposure to samples by using the luminometer Microtox®M500. Samples were considered toxic when 50% reduction (EC_50_) of the bioluminescence (vs. control) was obtained.

#### 2.4.2 Phytoplankton

The growth inhibition tests of the marine diatom *P. tricornutum,* and the freshwater green alga *R. subcapitata* were performed according to ISO 10253 (ISO, 2016) and to OECD 201 (2011), adapting the protocol to the use of 24-well plates (Lukavský and Simmer, 2001).

The tests were carried out in triplicate. The three algal species were exposed to Aerodisp W740X (1.41, 14, 141 mg/L), Parsol TX (0.0001, 0.001, 0.01, 0.1, 1, 12.5, 25, 50), and cream leachates (100% - undiluted leachates, 16.6% - diluted leachates), namely, blank cream, Aerodisp W740X cream and Parsol TX cream for 72 h at 20 °C ± 0.5 °C with a 12:12 light:dark photoperiod and light intensity of 6,000–10000 lux. The negative and solvent control were performed by F/2 medium and ethanol, respectively. After 72 h, a Lugol’s solution was used to stop the algal growth. Then, algal cells were counted under an inverted microscope (Leitz Diavert, Germany) by using a haemocytometer (Bürker chamber). The growth inhibition percentage was then calculated by comparing the algal growth in the test solutions with that of the negative control.

#### 2.4.3 Zooplankton

Immobility and swimming speed alteration (SSA) were evaluated in the larval stage of the marine crustaceans *Amphibalanus amphitrite* (II stage nauplii) and *A. franciscana* (Instar I larvae), obtained in laboratory conditions as reported in Garaventa et al. (2010) and Piazza et al. (2012). The bioassays on the barnacle *A. amphitrite* were performed according to UNICHIM standardized protocol published by the Italian regulatory authority (NU 2245/2012), while the bioassays with the brine shrimp *A. franciscana* with ISO testing procedure (ISO TS/20787). Tests were carried out in triplicates in multiwell plates with 1 mL of Aerodisp W740X (1.41, 14.10, 141, 1410 mg/L), Parsol TX (0.0001, 0.001, 0.01, 0.1, 1, 12.5, 25, 50 mg/L), and cream leachates (100% - undiluted leachates, 16.6% - diluted leachates), namely, blank cream, Aerodisp W740X cream, Parsol TX cream leachates and 10-15 organisms for each well. The plates were incubated for 48h at 20 ± 0.5 °C for *A. amphitrite* and 25 ± 0.5 °C for *A. franciscana*, in darkness. Subsequently, a stereo microscope was used to count immobile organisms. The percentage of immobility was calculated comparing the number of immobilized organisms to the negative control. Regarding the SSA, it was evaluated to register the crustacean’s movement for 3s in darkness by using the Swimming Behaviour Recorder system as described in Faimali et al. (2006). The average swimming speed (S) of each concentration was compared to the negative control to determine the percentage of SSA:

$$\text{SSA }\left(\%\right)=S\;\left(\text{Treated}‐\text{Control}\right)/\text{Control})\times 100$$

Immobility was also assessed in the freshwater crustacean *D. magna*, according to ISO 6341 (ISO, 1996) and the protocol provided by commercial kit Daphtoxkit FTM. The bioassays were carried out in triplicates in multiwell plates with 9 mL of the aforementioned test solutions and five daphnids for each well. The plates were incubated for 48h at 20 ± 0.5 °C in darkness. Then, immobile organisms (organisms with any movement for 15 s) were counted by using a stereomicroscope. Immobility percentages were calculated as described above for marine crustaceans.

### 2.5 Statistical analysis

Data of ecotoxicological tests are expressed as mean ± standard error (SE). Graphpad Prism five software was used for statistical analysis. The effect percentages deriving from three independent tests were interpolated using a nonlinear regression (log agonist vs. normalized response-variable slope) to calculate EC_50_s (Effective Concentration resulting into 50% algal growth inhibition, immobility or SSA in the exposed organisms). One-way ANOVA (Dunnett’s multiple comparison tests) was used to determine any significant differences between controls and treated samples (NOEC and LOEC values, *p < 0.05, **p < 0.01, ***p < 0.001 and ****p < 0.0001). For each species, statistically significant differences (**p < 0.01 and ***p < 0.001) were evaluated by Two-way ANOVA (Bonferroni post-tests) considering the “cream leachates” factor and the “percentages tested” factor.

## 3 Results

### 3.1 Evaluation of titanium concentration in leachates across ecotoxicological test media

As a preliminary step, titanium (Ti) concentration released from the different sunscreen formulations into the various aqueous media used for ecotoxicological testing was quantified. Table 1 reports the Ti concentration measured in leachates from the three sunscreen formulations-blank (without TiO_2_), and the two TiO_2_-containing formulations, Aerodisp W740X and Parsol TX-as determined by ICP-MS. In most media, Ti concentration was within the range of 1–8 μg/L, i.e., close to the instrument’s limit of quantification (LOQ = 10 μg/L). In contrast, Ti levels in the *Daphnia* medium (freshwater) were consistently below the LOQ for all formulations, including the blank, indicating negligible leaching or detection in that matrix. Four aqueous media were analyzed: two for marine organisms (seawater and marine algal culture medium) and two for freshwater organisms (freshwater algal medium and *Daphnia* medium). Overall, media with higher osmolarity-such as seawater and the marine algal medium-showed slightly higher Ti concentrations compared to freshwater media. This pattern may suggest enhanced TiO_2_ leaching and/or analytical interference due to increased salt content, which could influence particle dispersion or induce matrix effects. Among the quantifiable data, the freshwater algal medium exhibited the largest difference between the blank and the Aerodisp W740X formulation, while seawater showed the greatest difference between the blank and the Parsol^®^ TX formulation. These trends suggest that medium composition plays a role in modulating both the release and detectability of Ti from different TiO_2_-containing sunscreen products.

**TABLE 1:** Total titanium (Ti) concentration (µg/L) measured in undiluted cream leachates (blank cream, Aerodisp W740X cream, Parsol TX cream), prepared in the aquatic media (filtered seawater/freshwater and algal media).

			Ti concentration (µg/L)		
			Blank cream	Aerodisp W740X cream	Parsol TX cream
**Leachates**	**Marine**	**Seawater**	3.3	2.5	6.4
		**Algal medium**	5.1	5.5	6.3
	**Freshwater**	***Daphnia* medium**	0.28	1	0.3
		**Algal medium**	1.2	8.6	1.3

Overall, Ti concentrations were low across all tested media, suggesting limited release and availability of Ti from the formulations under the conditions used.

### 3.2 Ecotoxicological tests

**FIGURE 1:**
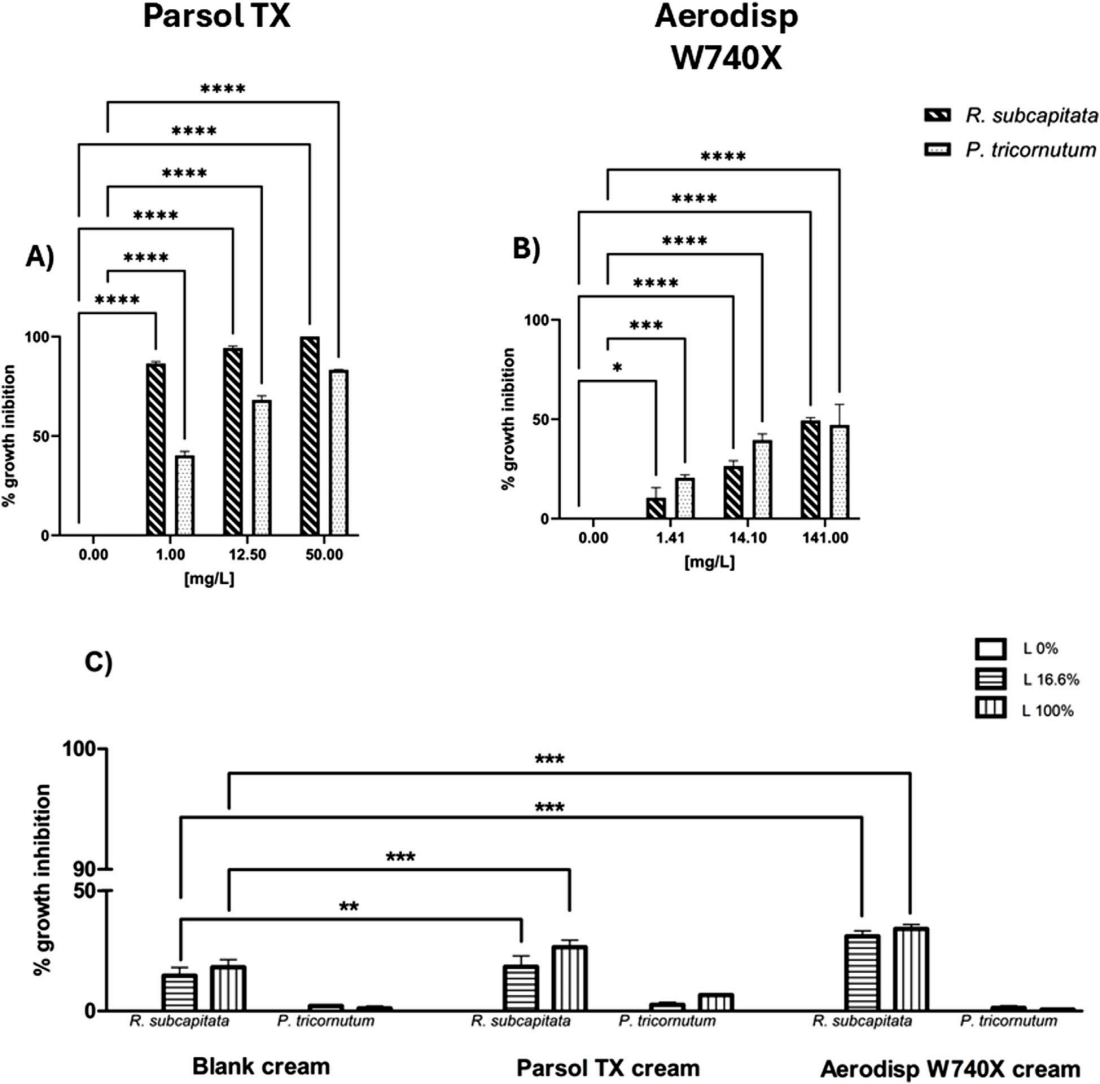
Growth inhibition of *R. subcapitata* (diagonal bars) and *P. tricornutum* (dotted bars) after 72 h exposure to: **(A)** Parsol TX, **(B)** Aerodisp W740X (active ingredients), **(C)** blank cream leachate, Parsol TX cream leachate, and Aerodisp W740X cream leachate. Data are mean ± SEM (n = 3). *p < 0.05, **p < 0.01, ***p < 0.001 and ****p < 0.0001 vs. control (One-way ANOVA + Dunnett for actives; Two-way ANOVA + Bonferroni for leachates). No asterisk indicates no statistical significance.

**FIGURE 2:**
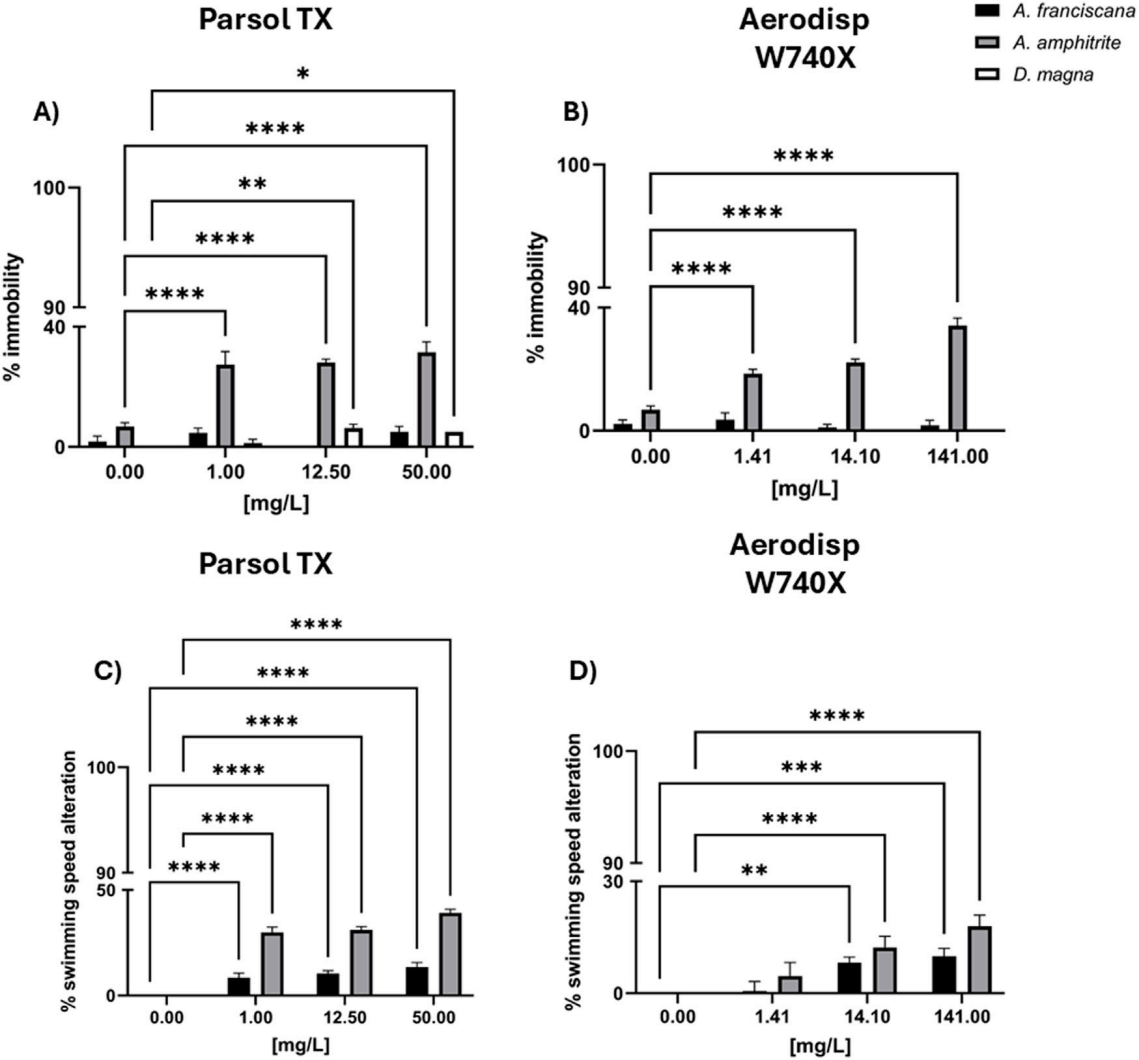
Immobility and swimming speed alteration in marine crustaceans *A. franciscana* (black bar) and *A. amphitrite* (grey bar), and in the freshwater crustacean *Daphnia magna* (white bar) after 48h exposure to different concentrations of Parsol TX **(A,C)** and Aerodisp W740X) **(B,D)**. Data are mean ± SE (n = 3). *p < 0.05, **p < 0.01, ***p < 0.001 and ****p < 0.0001 vs. control (One-way ANOVA + Dunnett for actives; Two-way ANOVA + Bonferroni for leachates). No asterisk indicates no statistical significance.

The ecotoxicological results from tests with aquatic species exposed to nano- and micro-sized TiO_2_ active ingredient and all cream leachates, are shown below. For clarity, all tested concentrations were used to calculate EC_50_ values (Tables 2, 3), while the most significant concentrations compared to the controls are shown in Figures 1, 2.

**TABLE 2:** EC_50_ values with confidence limits (95%), expressed in mg/L for Parsol TX and Aerodisp W740X calculated for phyto- and zooplankton species. For Parsol TX, the highest tested concentration was 50 mg/L; higher concentrations were not tested due to solvent toxicity limitations. For Aerodisp W740X, the highest testable concentrations for bacteria and phytoplankton were limited by sample opacity (milky solution). For zooplankton, the maximum tested concentration was 1410 mg/L.

				EC_50_ (95% CL)	
				Active ingredients	
		Exposure time	Endpoint	Parsol TX (mg/L)	Aerodisp W740X (mg/L)
**Bacteria**	*A. fischeri*	30m	Bioluminescence inhibition	>50	>1410
**Phytoplankton**	*P. tricornutum*	72h	Growth inhibition	0.38 (0.06-2.25)	>141
	*R. subcapitata*	72h	Growth inhibition	0.018 (0.007-0.45)	>141
**Zooplankton**	*A. amphitrite*	48h	Immobility	>50	84.6 (28.2-183.3)
			Behaviour	>50	239.7 (42.3-1381.8)
	*A. franciscana*	72h	Immobility	>50	>1410
			Behaviour	>50	>1410
	*D. magna*	48h	Immobility	>50	>1410

**TABLE 3:** EC_50_ values with confidence limits (95%) expressed as percentages for all creams leachates, calculated for phyto- and zooplankton species. ND = EC_50_ not determined within the tested concentration range. EC_50_ values were not reached at the highest percentage tested (100%, undiluted samples).

				EC_50_ (95% CL)		
				Creams		
		Exposure time	Endpoint	Blank cream (100%)	Parsol TX cream (100%)	AerodispW740X cream (100%)
**Bacteria**	*A. fischeri*	30m	Bioluminescence inhibition	ND	ND	ND
**Phytoplankton**	*P. tricornutum*	72h	Growth inhibition	ND	ND	ND
	*R. subcapitata*	72h	Growth inhibition	ND	ND	ND
**Zooplankton**	*A. amphitrite*	48h	Immobility	ND	ND	ND
			Behaviour	ND	ND	ND
	*A. franciscana*	72h	Immobility	ND	ND	ND
			Behaviour	ND	ND	ND
	*D. magna*	48h	Immobility	ND	ND	ND

#### 3.2.1 EC_50_ values and biological responses to TiO_2_ active ingredients and sunscreen leachates

To evaluate the potential impact of both the active ingredients and the leachates from the sunscreen formulations, several ecotoxicological endpoints across multiple aquatic species representing different trophic levels were assessed. Specifically, the following endpoints were measured: bioluminescence inhibition in the bacterium *A. fischeri*, growth inhibition in the phytoplankton species *P. tricornutum* and *R. subcapitata*, immobilization in the zooplankton species *D. magna*, while immobilization and behavioral responses in *A. amphitrite* and *A. franciscana*. Thus, Tables 2, 3 show the EC_50_ values obtained both for the active ingredients Aerodisp^®^ W740X and Parsol TX and all cream leachates.

For bacteria, no EC_50_ values were determined for both active ingredients within the range of concentrations tested. Concentrations higher than 141 mg/L for Aerodisp W740X could not be tested due to the opacity of the sample solution, while concentrations >50 mg/L for Parsol TX, were not tested due to solvent toxicity. Quantifiable EC_50_ values for Parsol TX were obtained only in phytoplankton species tested, with values of 0.38 mg/L for *P. tricornutum* and 0.0018 mg/L for *R. subcapitata*, indicating a higher sensitivity of primary producers to micro-TiO_2_. Conversely, for Aerodisp W740X, EC_50_ values were obtained only for the marine crustacean *A. amphitrite,* indicating a higher sensitivity of this species to the nano-sized TiO_2._

Additionally, no toxic effects–in terms of EC_50_s - were observed in any of the test species exposed to leachates from the blank or the TiO_2_-containing formulations.

#### 3.2.2 Bioluminescence inhibition in *A. fischeri* induced by TiO_2_ active ingredients and cream leachates

The inhibition of bioluminescence in *A. fischeri* was not significantly affected after 30 min exposure to various concentrations of Aerodisp W740X and Parsol TX, tested both as pure active ingredients and as cream leachates (Supplementary Figure S1). Both active ingredients exhibited a similar trend Supplementary Figure S1A), as well as for their corresponding cream leachates (Supplementary Figure S1B), showing 15% or 20% maximum effect in bioluminescence inhibition.

#### 3.2.3 Growth inhibition of freshwater and marine phytoplankton by TiO_2_ active ingredients and cream leachates

The growth inhibition percentage of the freshwater alga *R. subcapitata* and the marine diatom *P. tricornutum* after 72h of exposure to different concentrations of Aerodisp W740X and Parsol TX is shown in Figures 1A,B. Both active ingredients affected the growth of both algal species in a concentration-dependent manner (Figures 2A,B). Although no EC_50_ values could be estimated for Aerodisp W740X due to sample opacity at concentrations >141 mg/L, a significant inhibition of algal growth was observed in both freshwater (*R. subcapitata*) and marine (*P. tricornutum*) species starting from the concentration tested (1.41 mg/L; Figure 2B). Parsol TX showed marked toxicity in both algae, with significant effects observed at concentrations of 1 mg/L for both algal species.

The growth inhibition of both algal species was also evaluated in leachates of the three creams (blank cream, Aerodisp W70X cream and Parsol TX cream). Although, in *R. subcapitata*, the Parsol TX cream induced an algal growth inhibition of approximately 40% for both tested percentages (16.6%, 100%), no EC_50_ value was estimated for any cream leachate (Table 3).

Significant differences were found in the freshwater species *R. subcapitata* when the blank cream leachate was compared with the respective percentage (16.6%, 100%) of the cream with Aerodisp W70X or Parsol TX, highlighting a higher toxicity of the cream leachates containing the active ingredients (Two-way ANOVA, Bonferroni post-tests; Figure 2C). Conversely, no significant differences were observed for the marine species *P. tricornutum*.

Notably, the blank cream leachate affected the growth of both species, with a higher impact on *R. subcapitata,* indicating that formulation matrix can alter algal growth.

#### 3.2.4 Effects of sunscreen components on zooplankton immobility and behaviour

Figure 2 shows, the percentage of immobility in *A. franciscana*, *A. amphitrite*, and *D. magna*, and the percentage of swimming speed alteration (SSA) in the two marine crustaceans (*A. franciscan*a and *A. amphitrite*) after 48 h of exposure to different concentrations of Parsol TX (Figures 2A,C) and Aerodisp W740X (Figures 2B,D), SSA, defined as baseline swimming behaviour of the organisms, is a sensitive sublethal endpoint that can reveal early signs of physiological stress or neurotoxicity, even in the absence of mortality. No effects were recorded in marine and freshwater crustaceans after the exposure to the solvent control used for Parsol TX stock solution (<10% effect).

Both compounds negatively only influenced *A. amphitrite* immobility (2A,B) and swimming behaviour (2C,D). Specifically, after the exposure to Aerodisp W740X, significant percentages of immobilization (B) and swimming speed alteration (D) were observed in *A. amphitrite* from 1.41 mg/Land 14.1 mg/L, respectively, causing 100% effect at 1410 mg/L (data not shown). Significant ecotoxicological responses were also caused by Parsol TX at 1 mg/Lfor immobility and SSAmg/L), although only a 40% effect was recorded at the highest concentration (50 mg/L). No significant effects in terms of immobility and SSA were observed in *A. franciscana* nauplii and *D. magna* larvae (<10%) exposed to both compounds (Figure 2).

**FIGURE 3:**
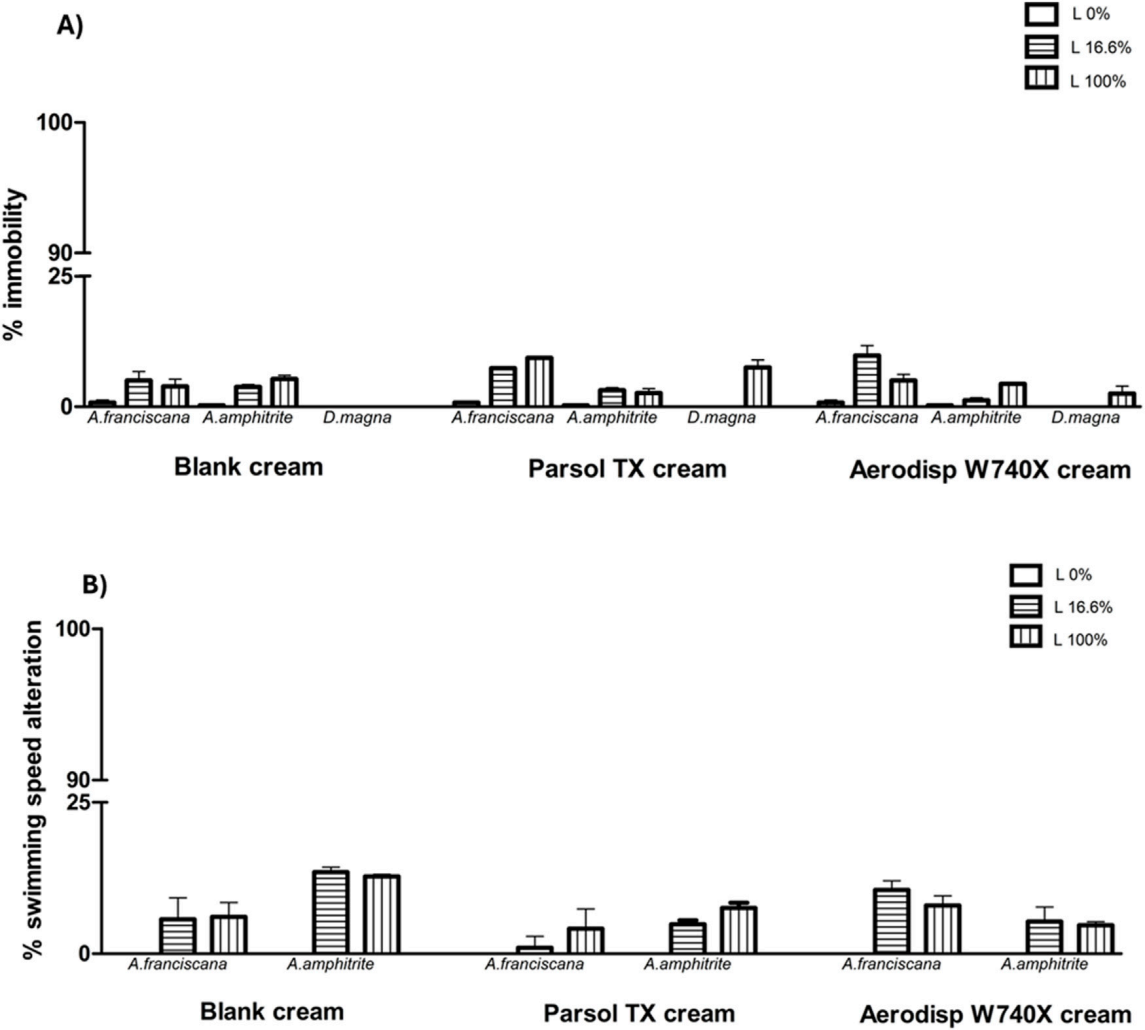
Immobility **(A)** and swimming speed alteration **(B)** in marine crustaceans *A. franciscana*, *A. amphitrite* and in the freshwater crustacean *Daphnia magna* after 48h exposure to 16.6% (diluted; horizontal line bar) and 100% (undiluted; vertical line bar) leachates of cream blank (without active ingredient), cream with cream with Parsol and Aerodisp W70X. No asterisk indicates no statistical significance (Two-way ANOVA, Bonferroni post-tests).

Figure 3 also shows the results of the three cream leachates on marine *(A. franciscana*, *A. amphitrite*) and freshwater (*D. magna*) zooplankton species in terms of immobility (A) and SSA (B). No cream leachates caused significant ecotoxicological effects (<20%; two-way ANOVA, Bonferroni post-test) at any dilution tested (16.6% and 100%).

## 4 Discussion

This study aimed to assess the potential ecotoxicological effects of nano- and micro-TiO_2,_ tested both as active ingredient alone (Parsol TX, Aerodisp W740X) and as components of sunscreen leachates containing the two different sizes of TiO_2_ (cream with Parsol TX, cream with Aerodisp W740X). For the first time, an *ad hoc* protocol simulating human immersion in the aquatic environment was applied to simulate a realistic exposure scenario. Additionally, to evaluate the ecotoxicological effects, we adopted a multi-species approach integrating both standard regulatory endpoints (mortality, immobility, growth inhibition) and sub-acute behavioural responses. This comprehensive approach is particularly relevant since standard assays, although essential for regulatory frameworks, may underestimate nano- and microparticles risk. Conversely, behavioural endpoints (e.g., altered swimming activity in crustaceans) are increasingly recognized as sensitive indicators of sublethal stress (Di Giannantonio et al., 2022; Gambardella et al., 2024). As noted by Hellou (2011), behavioural ecotoxicology may reveal pollutant effects that remain undetected by traditional assays, especially during short-term exposures. Therefore, our choice to include behavioural assays along with standardized tests reflects a significant strategy in the overall risk characterization of TiO_2_. Particularly, in this research, ecotoxicological endpoints (i.e., bacterial bioluminescence and algal growth inhibition, crustacean immobility and behaviour) were evaluated in selected marine and freshwater species belonging to three different levels of the aquatic food chains, to achieve a comprehensive assessment of TiO_2_ toxic effects across organizational levels.

In line with this multi-endpoint approach, the results revealed different sensitivity across the selected assays. Regarding bacteria, no significant inhibition of bioluminescence was observed after exposure to either nano- or micro-sized TiO_2_ (effect <20%; Supplementary Figure S1), as demonstrated by Heinlaan et al. (2008) who reported a median toxic effect only at high concentrations (EC_50_ > 20 g/L). Similarly, Lopes et al. (2012) found that 6 nm and ∼100 nm particle size TiO_2_ did not exert inhibitory effect in *A. fischeri.* Although bacterial bioluminescence test is widely adopted in ecotoxicological assessments due to inclusion in some international regulations, its sensitivity appears limited in specific contexts. As highlighted by Fekete-Kertész et al. (2017), most studies failed to detect any toxic effects of TiO_2_ at short exposure time (Froehner et al., 2000; García et al., 2011; Heinlaan et al., 2008; Strigul et al., 2009), raising concerns about the adequacy of this assay for evaluating the acute toxicity of nanomaterials. In this regard, the toxicity of nano- and micro-sized TiO_2_ was also assessed by using longer exposure times, by applying bioassays on aquatic crustaceans (48 h) and algae (72 h). Regarding phytoplankton, both Aerodisp W740X (nano- TiO_2_) and Parsol TX (micro- TiO_2_) induced concentration-dependent growth inhibition in freshwater and marine algae (Figure 1). However, Parsol TX exhibited higher toxicity, as shown by its lower EC_50_ values (0.018 mg/L for *R. subcapitata*, 0.38 mg/L for *P. tricornutum*). Although micro-TiO_2_ was coated with silica and dimethicone to improve its safety (Trivedi et al., 2025), in our study this coating may have contributed to the increased toxicity observed in algae. These results highlight the relevance of considering surface treatment, alongside particle size and UV filter concentrations, when evaluating the environmental risks of TiO_2_-based sunscreens. These findings align with those reported by Sendra et al. (2017), who also observed, based on EC_50_ values, a higher toxicity of microsized TiO_2_ compared to nanosized TiO_2_ in the freshwater alga *Chlamydomonas reinhardtii*. Nano- TiO_2_ is known to induce exo-polymeric substances (EPS) production, which could reduce its bioavailability by promoting agglomeration, limiting particle-cell contact, and trapping particles outside the cell (Khan et al., 2011). This mechanism could explain the reduced toxicity of nano-TiO_2_ compared to the micro- TiO_2_ observed in this study. This low toxicity is in line with the findings of Wang et al. (2016), who reported an EC_50_ of 167.7 mg/L and a LOEC of 20 mg/L, as similar high effective concentrations (141 mg/L and 14.1 mg/L) were also identified in both algal species in our research. However, these results regarding n-TiO_2_ differ from most previous findings, which have reported markedly higher toxicity levels, often with EC_50_ values in the range 10–100 mg/L in various marine and freshwater algal species (Aruoja et al., 2009; Hartmann et al., 2010; Miller et al., 2012; Clément et al., 2013; Li et al., 2015). Several studies have attempted to demonstrate the mode of action through which TiO_2_ could inhibit algal growth. One of the main ones concerns oxidative stress, in particular lipid peroxidation, with high levels of malondialdehyde (MDA), superoxide dismutase (SOD) and catalase (CAT) (Ma et al., 2013; Melegari et al., 2013). After exposure to nano-TiO_2_, Lin et al. (2012) reported a significant increase in MDA levels in *Chlorella* sp. In *Karenia brevis*, a severe reactive oxygen species (ROS) generation led to significant damage to intracellular organelles and the cell membrane, ultimately impairing algal growth (Li et al., 2015). On the other hand, Wang et al. (2008) highlighted the ‘shading effect’ caused by particle aggregation, which could reduce both the availability of active sites and the specific surface area. Furthermore, Schwab et al. (2011) provided evidence that micro and nano-TiO_2_ particles could penetrate algal cells and adhere to chloroplasts, thereby interfering with light absorption by chlorophyll and ultimately impairing photosynthetic activity and growth. In addition, TiO_2_ can also adsorb essential nutrients such as zinc and phosphorus from the growth medium, thereby reducing their bioavailability to algae (Kuwabara et al., 1986).

In several algal species, a severe reactive oxygen species (ROS) generation due to photoactivation (i.e., light, UV irradiation) led to significant damage to intracellular organelles and the cell membrane, ultimately impairing algal growth (Li et al., 2015; Roy et al., 2016; Baniamerian et al., 2020). On the other hand, Wang et al. (2008) highlighted the ‘shading effect’ caused by particle aggregation, which could reduce both the availability of active sites and the specific surface area. Furthermore, Schwab et al. (2011) provided evidence that micro and nano-TiO_2_ particles could penetrate algal cells and adhere to chloroplasts, thereby interfering with light absorption by chlorophyll and ultimately impairing photosynthetic activity and growth. In addition, TiO_2_ can also adsorb essential nutrients such as zinc and phosphorus from the growth medium, thereby reducing their bioavailability to algae (Kuwabara et al., 1986). The different toxicity found in the algae exposed to nano- or micro-sized TiO_2_ could be ascribed to all these mechanisms (i.e., photoactivation, shading effects) since a photoperiod was used; further investigations are required to elucidate the main factors contributing to algal toxicity.

Regarding zooplankton, two crustaceans out of three (*A. franciscana*, *D. magna*) were not affected - in terms of acute and behavioural responses - by nano and micro-TiO_2_ active ingredients or incorporated into creams by any concentrations (Figures 2, 3). Generally, *A. franciscana* and *D. magna* are considered among the most tolerant aquatic invertebrates to a wide range of environmental contaminants (Pinto and Zanette, 2023; Al-Shidi and Sulaiman, 2024). Our findings are in line with previous studies reporting the absence of toxic effects of nano- TiO_2_ in these aquatic invertebrates. Specifically, Ates et al. (2013) and Heinlaan et al. (2008) observed no toxic effects in *A. franciscana* and *D. magna*, respectively. To the best of our knowledge, no data on the effects of micro- and nanosized TiO_2_ on *A. amphitrite* nauplii are currently available in the literature. This makes the present research the first study ever carried out on this species. Thus, *A. amphitrite* was the only species to exhibit significant sensitivity to both compounds. TiO_2_ is known to induce strong ROS production in crustaceans, which could compromise membrane integrity through lipid peroxidation. This oxidative stress may subsequently damage proteins and nucleic acids, impair cellular antioxidant defenses, and ultimately lead to cell death (Bhuvaneshwari et al., 2017; Thiagarajan et al., 2022). Kim et al. (2010) also demonstrated that the mortality induced by TiO_2_ nanoparticles was attributed to the oxidative stress, which was indirectly related to the oxidative stress markers, such as MDA, CAT and GSH. Oxidative stress and immune system impairment were also found in the marine crustacean *Moina mongolica* after short-term exposure to nano-TiO_2_ (Huang et al., 2022).

Further investigations focusing on oxidative stress biomarkers and molecules involved in the immune response in barnacle nauplii will help to clarify the mechanisms responsible for the toxicity of micro- and nano-sized TiO_2_ active ingredients.

To better understand the ecological relevance of our findings, we compared the effect concentrations determined in this study with TiO_2_ concentrations measured in the aquatic environments. The latter are frequently detected in the aquatic environment at concentrations varying between freshwater systems (6.5–86 μg/L; Neal et al., 2011; Shi et al., 2016) and seawater (37–900 μg/L Tovar-Sánchez et al., 2013; Labille et al., 2020). The toxic effects measured in the present study on phyto- and zooplankton (Table 1) overlap the environmental levels, suggesting a potential ecotoxicological risk of TiO_2_ active ingredients in both freshwater and marine ecosystems.

Due to its strong UV radiation blocking properties, TiO_2_ is widely used as an active ingredient in sunscreen formulations (Foltête et al., 2011). However, similar to other cosmetic formulations, sunscreens can enter aquatic environments through direct release during recreational water activities or indirectly via treated wastewater discharged into natural water bodies. The level of TiO_2_ release from topical formulations following dermal application can vary considerably, depending on both the formulation type and environmental conditions. In studies employing realistic exposure scenarios, TiO_2_ release ranged from approximately 5% to just over 30% over a 48-h period (Botta et al., 2011). In another study, estimated release rates ranged from 10% to 22% for cream-based sunscreens and from 40% to 46% for milk-based formulations applied to pig skin after 120 min of immersion in continuously agitated water (Jeon et al., 2016). Chemical analyses revealed generally low concentrations of Ti released in freshwater and seawater from the tested formulations (Table 1). Considering that the highest expected concentration of Ti, based on the amount of cream applied (72 mg) and the declared TiO_2_ content in the formulations (5%), was approximately 3.6 mg/L, the measured values (<10 μg/L) indicate limited leaching of Ti into the aqueous media, independently from water composition (i.e., algal medium, freshwater, seawater). At such low concentrations, it was not possible to determine the particle size distribution by DLS, as also confirmed by TiO_2_ quantification through ICP-MS analysis. This limitation prevented a direct evaluation of the influence of water matrix and ionic strength on particle aggregation and behaviour.

In this study, we report for the first time the absence of toxicity in terms of EC_50_ and LC_50_ values-when comparing micro- and nano-TiO_2_ based sunscreens to the cream blank across all aquatic organisms. However, a significant growth inhibition between TiO_2_ based-sunscreens and cream blank was found in freshwater algae. This result cannot be ascribed to Ti release in the aquatic medium, since a similar Ti content was measured in all samples at low levels (<9 μg/L; Table 1). Thus, the significant effect found in freshwater algae rather than in marine ones may be due to the behaviour of TiO_2_ in the two different aquatic environments. Indeed, several studies report the aggregation of TiO_2_ in both freshwater and seawater (Brunelli et al., 2013; Gambardella et al., 2024); nevertheless, based on a comparison of these studies, aggregation in seawater appears to lead to larger agglomerates due to its higher ionic strength. These findings could explain the highest toxicity observed in freshwater algae, as low aggregation in freshwater could increase the bioavailability of TiO_2_ and consequently, lead to stronger growth inhibition.

All zooplankton species exhibited low sensitivity to the cream leachates, with no significant effects observed on immobility or swimming behaviour. These observations further support the hypothesis that Ti, after being incorporated into a formulation and released at environmentally relevant concentrations, exerts lower ecotoxicological pressure on zooplankton compared to the active ingredient alone.

## 5 Conclusion

Here, we propose a leachate-based methodology for sunscreens combined with a multi-species and multi-endpoint ecotoxicological approach to evaluate the toxicity of TiO_2_. For the first time an *ad hoc* protocol simulating a realistic exposure scenario based on human immersion in an aquatic environment was applied, thereby enhancing the environmental relevance of the testing conditions and addressing a critical gap in the assessment of contaminants released from consumer products. Our results demonstrate the toxicity of UV filters as micro- and nano-TiO2 active ingredients towards marine and freshwater zooplankton, whereas no significant effects were observed for the sunscreen formulations. Considering the potential toxicity of the active ingredients, future studies should also address phototoxicity. Since the specificity of toxicity is linked to sunscreen design and realistic exposure conditions, in our research we considered the toxicity of individual active ingredients as well as that of the cream formulations. The latter were tested using a protocol specifically designed to reproduce realistic environmental exposure, where effective concentrations are generally much lower due to dilution. This finding highlights the importance of evaluating both ingredient-specific toxicity and formulation design to better predict ecotoxicological effects in real-world scenarios. Beyond regulatory implications, these findings also align with the One Health perspective, recognizing the interconnectedness between human wellbeing, environmental protection, and ecosystem health. In this context, the cosmetic industry is increasingly integrating environmental safety into product innovation, a particularly relevant aspect for sunscreens that may be directly released into marine water. Our work therefore not only addresses a scientific and regulatory gap but also supports the broader shift towards sustainable and environmentally responsible cosmetic formulations.

## Data Availability

The datasets presented in this article are not readily available because Data will be available upon request. Requests to access the datasets should be directed to roberta.nugnes@ias.cnr.it.
