# Supplementary material for: Comprehensive methodology for standardized ecotoxicological assessment of TiO2-based sunscreen leachates in aquatic environment
Source: Front Toxicol. 2025 Nov 3;7:1686954. doi: 10.3389/ftox.2025.1686954 (PMC12620257; doi:10.3389/ftox.2025.1686954)
Supplement: Supplementary file 1 [file Supplementaryfile1.docx]

Supplementary Material

# Supplementary Figure

**Supplementary Figure 1.** Inhibition of bioluminescence in *A. fischeri* after 30 minutes of exposure to Aerodisp W740X and Parsol TX. Panels show dose-response curves for (A) active ingredients and (B) cream leachates derived from formulations containing these actives. The blank cream leachate (formulation without TiO_2_) is also included for comparison. Data represent mean ± standard error of the 3 replicates. No asterisk indicates no statistical significance by One-way ANOVA, Dunnett’s multiple comparison test for active ingredients and Two-way ANOVA, Bonferroni for cream leachates.
